# Lack of Renal 11 Beta-Hydroxysteroid Dehydrogenase Type 2 at Birth, a Targeted Temporal Window for Neonatal Glucocorticoid Action in Human and Mice

**DOI:** 10.1371/journal.pone.0031949

**Published:** 2012-02-16

**Authors:** Laetitia Martinerie, Eric Pussard, Geri Meduri, Anne-Lise Delezoide, Pascal Boileau, Marc Lombès

**Affiliations:** 1 INSERM, U693, Le Kremlin-Bicêtre, France; 2 Univ Paris-Sud 11, Faculté de Médecine Paris-Sud, UMR-S693, Le Kremlin-Bicêtre, France; 3 Service d'Endocrinologie et Maladies de la Reproduction, Assistance Publique-Hôpitaux de Paris, Hôpital de Bicêtre, Le Kremlin-Bicêtre, France; 4 Service de Génétique Moléculaire, Pharmacogénétique et Hormonologie, Assistance Publique-Hôpitaux de Paris, Hôpital de Bicêtre, Le Kremlin-Bicêtre, France; 5 Service de Biologie du Développement, Assistance Publique-Hôpitaux de Paris, Université Paris-Diderot, Hôpital Robert Debré, Paris, France; 6 PremUp Foundation, Paris, France; 7 Service de Pédiatrie et Réanimation néonatale, Univ Paris-Sud 11, Assistance Publique-Hôpitaux de Paris, Hôpital Antoine Béclère, Clamart, France; Hôpital Robert Debré, France

## Abstract

**Background:**

Glucocorticoid hormones play a major role in fetal organ maturation. Yet, excessive glucocorticoid exposure in utero can result in a variety of detrimental effects, such as growth retardation and increased susceptibility to the development of hypertension. To protect the fetus, maternal glucocorticoids are metabolized into inactive compounds by placental 11beta-hydroxysteroid dehydrogenase type2 (11βHSD2). This enzyme is also expressed in the kidney, where it prevents illicit occupation of the mineralocorticoid receptor by glucocorticoids. We investigated the role of renal 11βHSD2 in the control of neonatal glucocorticoid metabolism in the human and mouse.

**Methods:**

Cortisol (F) and cortisone (E) concentrations were measured in maternal plasma, umbilical cord blood and human newborn urine using HPLC. 11βHSD2 activity was indirectly assessed by comparing the F/E ratio between maternal and neonatal plasma (placental activity) and between plasma and urine in newborns (renal activity). Direct measurement of renal 11βHSD2 activity was subsequently evaluated in mice at various developmental stages. Renal 11βHSD2 mRNA and protein expression were analyzed by quantitative RT-PCR and immunohistochemistry during the perinatal period in both species.

**Results:**

We demonstrate that, at variance with placental 11βHSD2 activity, renal 11βHSD2 activity is weak in newborn human and mouse and correlates with low renal mRNA levels and absence of detectable 11βHSD2 protein.

**Conclusions:**

We provide evidence for a weak or absent expression of neonatal renal 11βHSD2 that is conserved among species. This temporal and tissue-specific 11βHSD2 expression could represent a physiological window for glucocorticoid action yet may constitute an important predictive factor for adverse outcomes of glucocorticoid excess through fetal programming.

## Introduction

Glucocorticoid hormones play a critical role in promoting maturation of fetal organs essential for neonatal adaptation to extrauterine terrestrial life. Numerous studies have underlined the importance of glucocorticoids in fetal lung development. It is well established that antenatal administration of corticosteroids in women at risk of preterm delivery prevents neonatal respiratory distress syndrome [1]. Moreover, *via* the activation of the glucocorticoid receptor (GR), a transcription factor, glucocorticoids are able to stimulate the expression and activation of the epithelial sodium channel in lungs [2], crucial for pulmonary fluid resorption at birth [3]. However, excessive glucocorticoid exposure *in utero* has numerous harmful effects. It reduces fetal growth [4] and is associated with increased susceptibility to the development of hypertension [5]–[7], glucose intolerance [8] and anxiety related disorders in adulthood [9]. These deleterious effects have been related to epigenetic modifications during fetal programming [10], [11].

Transfer of maternal glucocorticoids to the fetus is controlled mainly by a placental functional barrier : the enzyme 11-beta-hydroxysteroid dehydrogenase type 2 (11βHSD2). This enzyme metabolizes active glucocorticoids into inactive 11-keto compounds i.e cortisol (F) into cortisone (E) in humans or corticosterone (B) into 11-dehydrocorticosterone (A) in rodents, while it has almost no effect on the metabolism of synthetic glucocorticoids (betamethasone and dexamethasone) or aldosterone [12]. Its capacity to inactivate glucocorticoids is extremely powerful in the placenta and increases during gestation [13], protecting the fetus from excessive impregnation by maternal glucocorticoids. Indeed, reduced placental 11βHSD2 activity has been associated in humans and mice with intra-uterine growth retardation [13], [14], and preeclampsia [15], [16].

11βHSD2 is also expressed by various organs aside from the placenta. Particularly, it has been shown to colocalize with the mineralocorticoid receptor (MR) in aldosterone-sensitive epithelial tissues [17], [18] where it is a key element of mineralocorticoid selectivity [19] protecting MR from illicit occupancy and activation by overwhelming cortisol concentrations [20]–[23].

Many studies have focused on placental 11βHSD2 activity, but little is known about its expression and function in the neonatal kidney. Significant levels of 11βHSD2 mRNA have been detected in mouse embryonic kidneys at various developmental stages associated with a decrease of mRNA expression near term, by *in situ* hybridization or northern blot analyses [24]–[26]. Similar results have been found in human fetal kidneys with an onset of 11βHSD2 expression early during gestation [17], [27]. However, 11βHSD2 renal protein expression and activity at birth have never been reported. The phenotype observed in 11βHSD2 knock-out newborn mice could be related to the lack of placental expression as well as to the absence of renal expression. The susceptibility to hypertension after fetal exposure to high levels of glucocorticoids could also be related to direct renal effects of glucocorticoids [11].

We have previously demonstrated that both mouse and human newborns have very low renal MR expression at birth [28]. Therefore, we hypothesize that the 11βHSD2 enzyme is either absent or below detectable threshold in the newborn kidney, since MR protection is not required. In order to verify this hypothesis, we measured cortisol (F compound) and cortisone (E compound) concentrations in maternal plasma, umbilical cord blood and in human newborn urine. We report the first evaluation of renal neonatal 11βHSD2 activity, by comparing the F/E ratio between plasma and urine in human newborns. Subsequently, we assessed 11βHSD2 activity at various developmental stages in mouse kidneys. We have also analyzed renal 11βHSD2 mRNA and protein expression during gestation, in both species, using quantitative RT-PCR and immunohistochemistry, which has not been reported to date. Our results demonstrate a lack of renal 11βHSD2 enzymatic activity at birth, related to a low renal expression, which parallels the relatively low fetal plasma cortisol levels measured in human newborns.

## Materials and Methods

### Patients

Patients have been previously described [29]. In brief, 44 healthy mother-neonate couples were included in the study. Singleton pregnancies were uncomplicated, and led to the birth of full-term healthy eutocic newborns, which were enrolled in the study. Informed and written consents were obtained for all patients. Written parental consent was obtained for all newborn testing. The study was approved by the local ethical committee (CCPPRB : Comité Consultatif Pour les Personnes en Recherche Biomédicale, Hôpital Antoine-Béclère, Clamart, France), and was conducted in regards to the Declaration of Helsinki's guidelines. Plasma and urine were collected from fifty healthy female adults, after obtention of informed and written consents, and used as controls.

### Plasma and urinary samples

Collection and conservation of maternal plasma samples and neonatal plasma and urine samples have been described previously [29]. Maternal blood samples were collected just before delivery, during preanalgesia peridural checkup. Neonatal blood samples were obtained from umbilical cord vein at birth. Single-spot urinary samples were collected on the day of birth from a gauze compress settled in the newborns diapers. This simple, non invasive, original method has already proven its efficacy for the measurement of aldosterone concentrations [29]. We did not perform 24-hours urinary collection in newborns for evident ethical reasons.

### Hormonal and biochemical analyses

Simultaneous determination of cortisol and cortisone was conducted by using a high performance liquid chromatography of maternal plasma, umbilical cord blood and urine samples as previously described [30]. For blood samples, steroids were extracted from plasma with dichloromethane. For urine analysis, steroids were extracted with dichloromethane and purified on accubond II Octyl cardridges 100 mg (Agilent Technologie, Interchim, Montluçon, France). 6α-methylprednisone was used as an internal standard. After evaporation, the residue was reconstituted in mobile phase for HPLC injection. The Waters HPLC system (515 pump, Wisp 717 plus autosampler and a UV 486 absorbance detector at 254 nm) was equipped with a Symmetry Shield 5 µm C8 reverse phase column (Waters, Saint Quentin, France). The mobile phase was a 55% methanol in water solution at a flow rate of 0.8 ml/min. The detection limit for both steroids was 1–2 ng/ml and the intra- and inter-day assay variations were less than 10%. Extraction recoveries of both steroids were more than 90% in plasma and 70% in urine. The detection was linear over the range of 10 to 1000 ng/ml. Urinary creatinine concentrations were measured with automat Modular P900 using a rate-blanked Jaffé based method (Roche Diagnostics, France). Urinary cortisol and cortisone concentrations were normalized by urinary creatinine levels.

### Mouse kidney samples

Mice were cared according to the Guide for the Care and Use of Laboratory Animals published by the US National Institutes of Health (NIH Publication No. 85-23, revised 1996). The animal facility was granted approval (N° B94-043-12), with an authorization to experiment on living animals (75–978, ML) given by the French Administration (Prefecture du Val de Marne, Direction départementale des services vétérinaires du Val de Marne). Wild type mouse kidneys were collected at 17.5 days of gestation (E17.5) (embryonic day 0.5 being the day of the plug), at birth (D0.5) and on postnatal day 8 (D8.5). Kidneys were snap-frozen in liquid nitrogen for quantitative RT-PCR analyses. Animal experiments and housing were conducted in accordance with the guidelines of the animal ethics committee of the Ministère de l'Agriculture (France).

### Human kidney samples

Snap-frozen fetal kidney samples were obtained from the Foetopathology Department of Robert Debre Hospital. They originated from *in utero* fetal deaths with rapid delivery and autopsy within 24 to 36 hours *post mortem*. Nineteen samples from fetuses aged 14–40 gestational weeks were used for qPCR quantification of renal 11βHSD2 expression. Parental informed and written consents were collected for all samples, after declaration of the study to the French Biomedical Agency (Decree 003812, 09/22/2006).

### Quantitative real-time RT-PCR

Two micrograms of total RNA isolated from frozen human and mouse kidney samples were submitted to reverse-transcription, using 200 U of reverse transcriptase (Superscript II; Invitrogen). Quantitative PCR were subsequently performed using 100 ng cDNA with qPCR Mastermix Plus for Sybr Green I (Eurogentec) and 300 nM of specific 11βHSD2 primers. Oligonucleotid sequences were as follows:

Mouse 11βHSD2 sense: 5′-AACCTCTGGCAGAAACGCAAG-3′ and antisense: 5′-GGCATCTACAACTGGGCTAAGG-3′

Human 11βHSD2 sense: 5′-CTGGACTCCATGGGCTTCAC-3′ and antisense: 5′-TGAACTCTAGCACGCGGCTAA-3′

Reaction parameters were carried out on an ABI 7300 Sequence Detector (Applied Biosystems). For standards preparations, amplicons were subcloned into pGEMT-Easy plasmid (Promega, Madison, WI). Standards were sequenced in order to confirm their identity. Serial dilutions of the linearized plasmids were used to generate standard curves. Standard and samples values were determined in duplicate from at least three different experiments. Relative expression for a given sample was expressed as the ratio between the expression of the specific gene in attomoles normalized by ribosomal 18S expression in femtomoles, which was used as an internal control.

### *11βHSD2* assay

Mouse kidneys at different developmental stages were collected and snap-frozen in dry-ice. Samples were homogenized and sonicated in ice-cold 0.1 M PBS pH 7.5. After centrifugation at 10 000 *g* for 30 min, the resulting supernatant was assayed for protein concentration using the BCA protein assay kit (Pierce, Rockford, IL, USA) with BSA as a standard. C11 dehydrogenase activity was determined by measuring the rate of conversion of ^3^H-cortisol to produce ^3^H-cortisone as previously described [31]. Fifty or 100 µg of protein homogenate in PBS buffer and 1 nM tritium-labeled cortisol in methanol were incubated 120 min at 37°C with or without addition of 1 mM NAD and 10^−6^ M carbenoxolone. Steroids were extracted with ice-cold ethyl-acetate. After evaporation of the organic phase, cortisol and cortisone were separated on the HPLC system described above and monitored by an ultraviolet absorbance detector and a Berthold LB 513 scintillation counter to detect tritiated steroids. Calibration was carried out with standard calibration curves of labeled cortisone. The rate of F to E conversion was expressed as the amount of picomoles of ^3^H-cortisone formed per mg protein per 2 hours.

### Immunohistochemistry

Neonatal and infantile human kidneys were selected from two pathology departments' collections according to the guidelines of the French Biomedical Agency. These samples originated from sudden infant deaths. Quality and integrity of the samples were verified by immunostaining with vimentin (as a control of appropriate formol fixation) and a low molecular weight cytokeratin (as a control of epithelial tubular cell integrity). Three different antibodies were used: polyclonal antihuman 11βHSD2 (Alpha Diagnostic International, San Antonio, Texas) at 15 µg/ml dilution, monoclonal anti-vimentin clone V9 (Biogenex Laboratories, San Ramon, CA) and anti-cytokeratin 19 antibodies (Progen, Queensland, Australia), at dilutions of 1∶100 and 1∶120 respectively. Immunohistochemistry was conducted as previously described [28]. Briefly, after paraffin removal, and antigen retrieval in Tris-EDTA pH 9 buffer at 85°C in a hot water bath for 2 h (for 11βHSD2) or in citrate buffer pH 6 in a microwave oven for 15 min (for vimentin and cytokeratin), slides were incubated overnight at 4°C with the appropriately diluted primary antibody or control immunoglobulins (rabbit and mouse IgG and control ascite fluid (Sigma)). Bound immunoglobulins were then revealed with a biotin-free immunostaining Kit (Immpress Reagent kit, Vector, Burlingame, California). Aminoethylcarbazol (DAKO, San Antonio, Texas) was used as a chromogen.

### Statistical analyses

Data are expressed as mean ± SEM. For qPCR analyses of 11βHSD2 expression in mouse kidneys, six samples from at least six different mice were used for each developmental stage. For human samples, results represent the mean of at least three independent experiments of three different reverse- transcribed samples, normalized using an internal calibrator. For 11βHSD2 assay, results represent the mean of three independent experiments for each developemental stage and each condition. Statistical analyses were performed using computer software Prism 4 (GraphPad Software, San Diego, CA). Non parametric Mann-Whitney tests or unpaired t-tests were used to compare two parameters. Significant threshold was determined at 0.05.

## Results

Forty-four mother-neonate couples were included in the study. Forty-three maternal blood samples and 44 umbilical cord blood samples were obtained and analyzed for plasma cortisol (F) and cortisone (E) levels. Urinary samples were collected from fourteen of these neonates, allowing quantification of F and E excretion levels at birth.

### Placental 11βHSD2 is extremely active at the end of gestation

**Figure 1:**
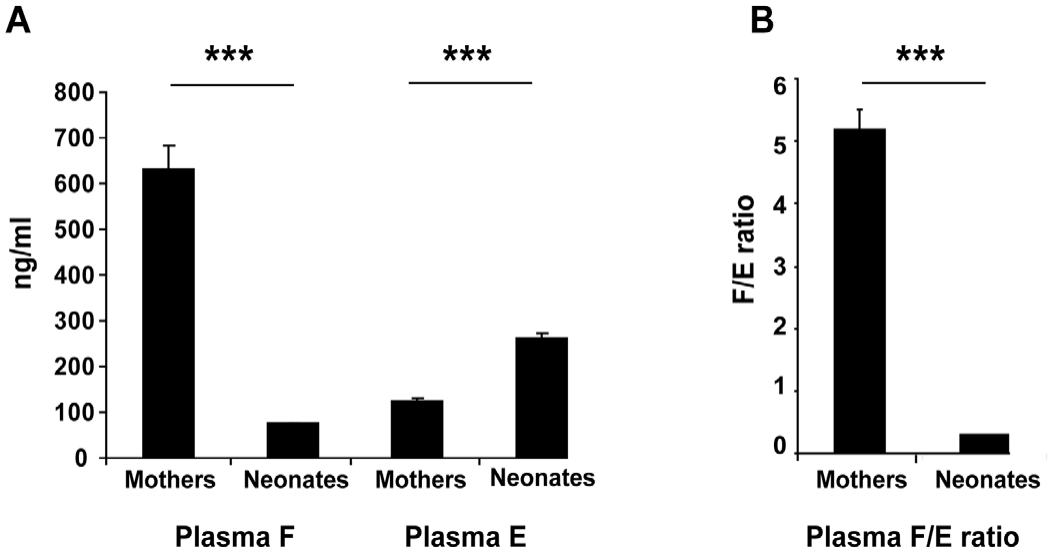
Plasma cortisol (F) and cortisone (E) levels, and F/E ratio in mothers and their neonates. A: Comparison of plasma cortisol and cortisone levels between mothers and neonates. Results for each parameter are expressed in ng/ml, as means ± SEM of the 43 values obtained from the mother-neonate couples. Plasma cortisol levels are significantly higher in mother than in neonates (*** *p*<0.001), and on the contrary, plasma cortisone levels are significantly lower (*** *p*<0.001). B: Comparison of the F/E ratio between maternal and neonatal plasma. The F/E ratio, which corresponds to cortisol metabolism by the 11βHSD2 enzyme, is significantly higher in mothers than in neonates (*** *p*<0.001). These results reflect a strong activity of the placental 11βHSD2.

We first compared plasma E and F levels between mothers and their neonates (Fig. 1). As observed in Figure 1A, maternal plasma F levels are significantly higher than neonatal levels (630±51 *vs* 75±6 ng/ml, respectively, *p*<0.0001). Conversely, cortisone levels are significantly lower in mothers than in their newborns (123±7 vs 260±14 ng/ml respectively, *p*<0.0001). This maternal-fetal gradient indicates that cortisol, present in high concentrations in maternal plasma, does not freely cross the placental barrier without metabolic conversion. Indeed, when we compared F/E ratio in both maternal and umbilical cord blood, we found a striking difference with a higher ratio in mothers than in newborns (5.2±0.30 *vs* 0.3±0.02, *p*<0.0001) in which E concentrations largely excedeed that of F (Fig. 1B). These results suggest that maternal cortisol is metabolized into cortisone by the placenta, afterwhich cortisone crosses the placental barrier into the fetal circulation. This could explain why high levels of cortisone are detected in newborns plasma. These results reflect the strong enzymatic activity of placental 11βHSD2, which persists in late gestation.

### Weak activity of renal 11βHSD2 in newborns

**Figure 2:**
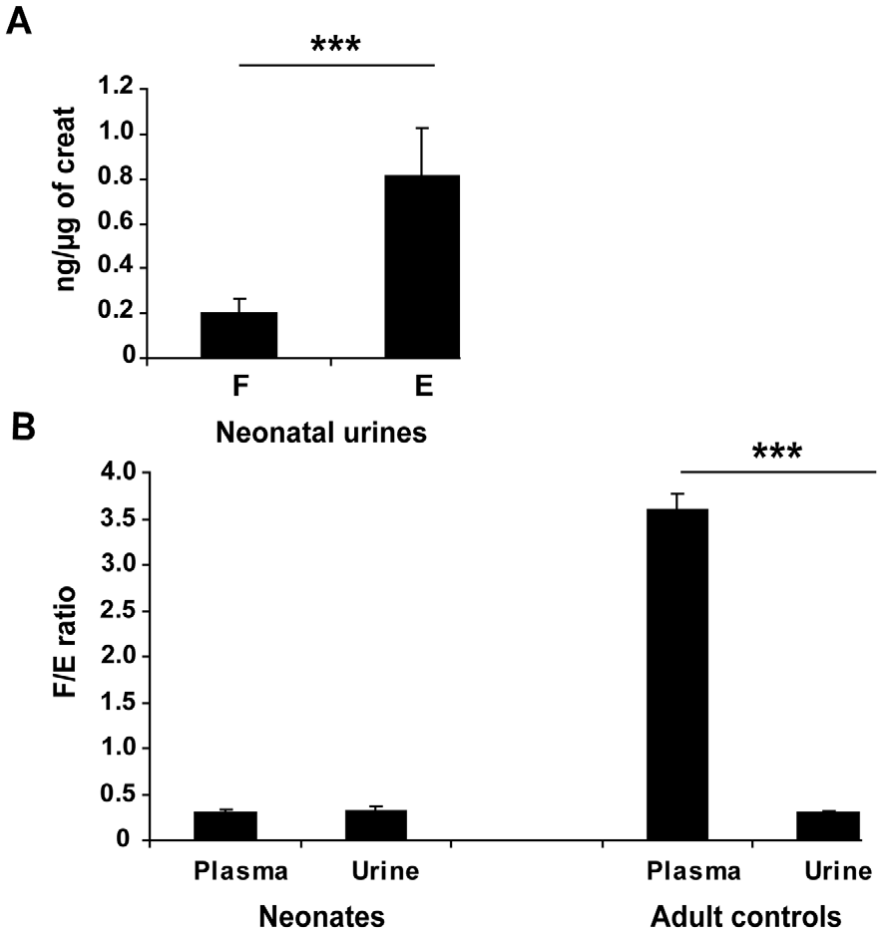
Urinary cortisol (F) and cortisone (E) concentrations and F/E ratio, in full-term newborns. A: Comparison between F and E in neonatal urine. Results are expressed in ng/µg of urinary creatinine concentration, as means ± sem of the 44 values obtained in neonates. Cortisol levels are significantly lower than cortisone levels (*** *p*<0.001). B: Comparison between plasma and urinary F/E ratio in neonates and in 50 healthy control adults. No difference was observed between the F/E ratio in plasma and urine at birth, which could reflect an inactivity of the neonatal renal 11βHSD2. On the contrary this enzyme is very active in adults, with an inversion of the F/E ratio in urine, significantly different from the plasma ratio (*** *p*<0.001).

As demonstrated in Figure 1B, plasma F levels are lower than E levels (*p*<0.0001) in newborns. These low levels of plasma cortisol may reflect the fetal adrenal biosynthesis [32]. High cortisone levels could either come from the mother *via* the placenta or from active cortisol metabolism by fetal 11βHSD2. To address this issue, we measured F and E concentrations in newborn urine collected onto a gauze compress (Fig. 2). This simple, non invasive method, suitable for steroid assessment [29], allowed us to obtain between 200 µl and 5 ml of urine. This is the first determination of urinary E and F concentrations at birth. Mean urinary F value was 75±6 ng/µg creatinine with median at 71 ng/µg creatinine. Mean urinary E value was 258±14 ng/µg creatinine with median at 225 ng/µg creatinine (Fig. 2A). Levels of urinary E were found significantly higher than those of urinary F (*p*<0.0001), which is normally expected and could reflect the activity of the renal 11βHSD2. To further investigate this activity, we compared the plasmatic and urinary F/E ratios of these neonates (Fig. 2B). Surprisingly, similar ratios were observed in these two compartments (0.31±0.02 *vs* 0.32±0.06, *p* = 0.95). In contrast, there was a strinking difference between the plasma and urinary F/E ratios in 50 healthy control adults (3.6±0.17 *vs* 0.3±0.01), demonstrating strong activity of renal 11βHSD2 in mature kidneys. These results therefore suggest that, at variance with adults, renal 11βHSD2 activity is very low in human newborns.

### 11βHSD2 mRNA expression and activity during mouse renal development

**Figure 3:**
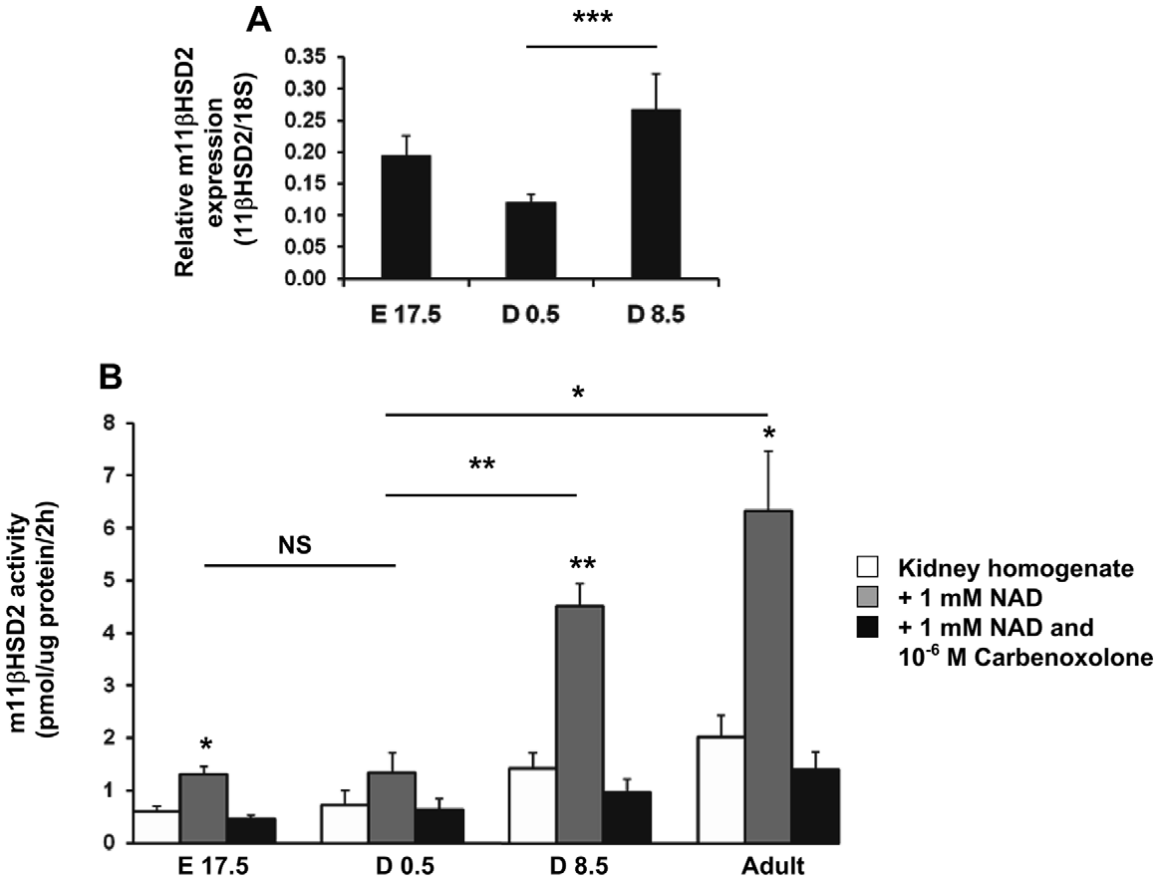
11βHSD2 mRNA expression and activity throughout renal development in mice. A: Relative mRNA expression in mice was determined using qPCR at various developmental stages, as follows : E 17.5, D 0.5 and D 8.5 E: Embryonic day, D: Postnatal day. Results, expressed as the ratio of attomoles of specific gene per femtomole of ribosomal 18S, correspond to mean ± SEM (E17.5 n = 9, D0.5 n = 17, D8.5 n = 6). *** *p*<0.001. B: Renal 11βHSD2 activity was determined at the same developmental stages and in adults, by measuring the rate of conversion of ^3^H-cortisol into ^3^H-cortisone, under three conditions : basal, with NicotinAmid Dinucleotid (NAD), with NAD and carbenoxolone, a specific 11βHSD2 inhibitor. Results, expressed as the ratio of picomoles of enzymatic activity after 2 hours per micrograms of protein, represent the mean ± SEM of three independent experiments for each developmental stage and each condition. * *p*<0.05; ** *p*<0.01, NS : Not Significant.

We have previously demonstrated that the mineralocorticoid signaling pathway follows a similar expression pattern both in mice and human, with a low MR mRNA and protein expression at birth and a postnatal up-regulation in both species [28]. Based on the MR expression profile, we wondered if this low renal 11βHSD2 activity in human newborns was conserved in the mouse. To further explore this hypothesis and investigate the underlying mechanisms, we quantified 11βHSD2 renal mRNA levels using quantitative RT-PCR and measured enzymatic activity by evaluating the rate of conversion of ^3^H-cortisol to ^3^H-cortisone throughout mice renal development. Renal organogenesis in the mouse starts at E8 and ends postnatally at D8. We have previously demonstrated that renal MR expression peaks at E17.5, is low at birth, and increases to reach a steady state at D8 in the postnatal period, while GR renal expression stays high throughout the perinatal period [28]. 11βHSD2 mRNA levels and enzymatic activity were therefore quantified at three developmental stages in the mouse : E17.5, D0.5 and D8.5, and in adult mice (Fig. 3A and 3B). We demonstrate that, mirroring MR, 11βHSD2 mRNA expression is low during gestation and especially at birth (0.12 amol/fmol 18S), before increasing significantly by 2-fold in the postnatal period, following kidney maturation (Fig. 3A). Interestingly, 11βHSD2 enzymatic activity correlated with mRNA expression with no detectable activity at birth, followed by a drastic postnatal increase (Fig. 3B). This enzymatic activity is specifically NAD-dependant and is inhibited by carbenoxolone.

These results therefore reveal that, as in humans, renal 11βHSD2 activity is very weak or undetectable in newborn mice, owing to a low 11βHSD2 mRNA expression at birth.

### 11βHSD2 mRNA and protein expression during human renal development

**Figure 4:**
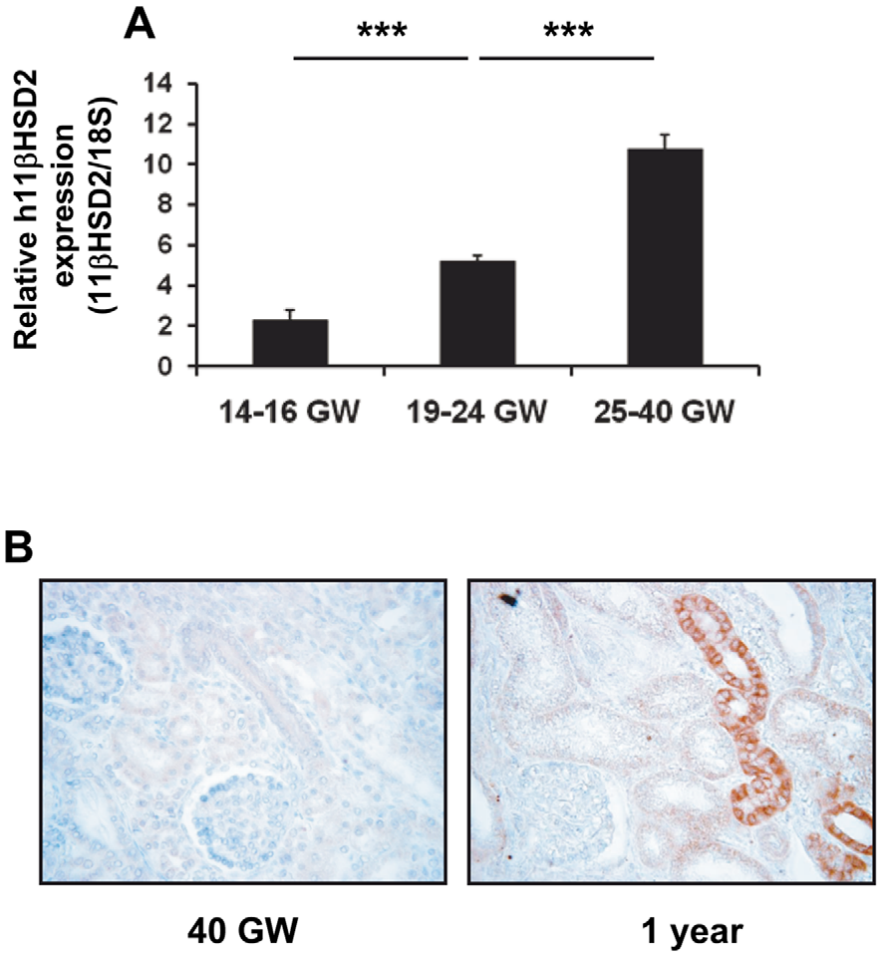
11βHSD2 mRNA and protein expression throughout renal development in humans. A: Relative mRNA expression in human fetal kidney samples at various gestational ages was determined using qRT-PCR at various gestational ages. Results are mean ± SEM of three independent determinations of 11βHSD2 mRNA expression, performed in triplicate for each sample. They are expressed as the ratio of attomoles of specific gene per femtomole of 18S, normalized by an internal calibrator. GW : gestational weeks. 14–16 GW n = 2, 19–24 GW n = 13, 25–40 GW n = 4. *** *p*<0.001. B: Immunodetection of the 11βHSD2 protein at 40 GW and at 1 year of age in human kidney samples. The 11βHSD2 protein is not detected in human kidney at birth whereas it is readily detected in the cytoplasm of cortical collecting duct cells at one year of age. Original magnification ×20.

To further investigate the possible underlying mechanisms of low renal 11βHSD2 activity in human newborns, we quantified mRNA levels by qPCR throughout gestation in 19 human kidney samples collected from fetuses aged 14 to 40 gestational weeks [33], and analyzed protein expression using immunohistochemistry in kidney samples from a newborn and a one year old infant (Fig. 4). Human renal organogenesis occurs between 5 and 36 GW, but human kidneys only reach full maturation at the end of the first year of life [28], [34]. We found that 11βHSD2 mRNA expression increases progressively and significantly (*p*<0.0001) throughout gestation (Fig. 4A), paralleling renal maturation and that this progression is likely to continue in the postnatal period. This was also observed in the mouse, with lower renal mRNA levels at birth than during the postnatal period. Indeed, no 11βHSD2 protein is detectable by immunohistochemistry in newborn kidney, whereas a specific immunolabeling is observed at one year of age in renal cortical collecting duct epithelium (Fig. 4B).

In summary, all results concur to demonstrate a very weak or absent renal 11βHSD2 enzymatic activity at birth, in relation to a low mRNA and protein expression, both in mice and humans.

## Discussion

This study represents the first demonstration of a weak or absent 11βHSD2 activity in the human newborn kidney. In human neonates, the ratio cortisol/cortisone reflecting the dynamics of the enzymatic conversion of glucocorticoids by 11βHSD enzymes is strictly identical between newborn plasma and urine, whereas in adult controls, it is clearly higher in plasma than in urine, emphasizing the strong activity of adult renal 11βHSD2. The conversion of cortisone to cortisol by 11-beta-hydroxysteroid dehydrogenase type 1 (11βHSD1) mainly occurs in the liver and is almost undetectable in the kidney in both mouse and human [25], [26], [35]. Thus, the steady state of F/E ratio between plasma and urine in human newborns relies rather on the absence of renal 11βHSD2 activity than on an equilibrium between renal 11βHSD2 and 11βHSD1 activities. This observation has been confirmed by direct measurement of 11βHSD2 activity in mouse kidney at various developmental stages. The enzymatic activity is very low in the prenatal and neonatal period and increases drastically postnatally. We have demonstrated that this weak activity is related to a low renal 11βHSD2 mRNA expression at birth both in mice and human which is concomitant with the absence of detectable protein in human kidney. Interestingly, others have demonstrated a specific 11βHSD2 expression in the fetal kidney both in mice [24]–[26] and human [17], [27] that contrasts with a low fetal enzymatic activity as demonstrated at E17.5 in our study and at E15 in [26], and could result from protein instability [36].

Therefore, renal 11βHSD2 follows a triphasic temporal expression profile, conserved among species that is reminiscent of that of the renal expression profile of other actors of the mineralocorticoid signaling pathway: the mineralocorticoid receptor and the alpha-subunit of the epithelial sodium channel (αENaC) [28], with a peak of expression during fetal life, a specific downregulation in the neonatal period and an up-regulation in the postnatal period. It could thus be hypothesized that all these molecular signaling partners follow a parallel maturation profile throughout renal development. On the other hand, is ensuring MR protection the only role of renal 11βHSD2 ? Moreover, could the absence of expression and activity of the enzyme in neonatal kidneys be particularly relevant during this specific developmental stage?

The weak renal 11βHSD2 activity contrasts with the strong placental enzymatic activity persistent in the prenatal period. An increased 11βHSD2 expression has been previously described in human placenta during the last trimester, associated with a decreasing F/E ratio in the amniotic fluid [37]. This heightened activity is necessary to protect the fetus from excessive glucocorticoid hormone exposure. Indeed, lack of placental 11βHSD2 activity leads to fetal and neonatal complications similar to those of glucocorticoid overexposure : reduced fetal growth [38] and susceptibility to development of hypertension and metabolic syndrome [7]. Human placental 11βHSD2 expression has been shown to be up-regulated by glucocorticoids *via* the GR, which represents an important safeguard mechanism [39]. Circulating cortisol levels at birth originate most notably from fetal adrenal biosynthesis as early as the second trimester of gestation [40] and/or the rise in 11beta-hydroxysteroid dehydrogenase type 1 expression in human fetal membranes near term [41], [42]. However, these concentrations are low and might therefore not be adequate for optimal and sustained stimulation of renal 11βHSD2 expression. Yet, they are sufficient to fully occupy the GR and activate the glucocorticoid signaling pathway [43]. Moreover, we have previously demonstrated that GR is expressed in the kidney at birth both at the mRNA and protein levels [28]. GR is detected in the nuclei of the cortical collecting duct cells, suggesting ligand-mediated activation of this receptor and functionality of the glucocorticoid signaling pathway. We could then wonder why the major regulator of glucocorticoid metabolism, 11βHSD2, is not expressed? Could this lack of expression represent a temporal window necessary for glucocorticoid activity? The combined lack of renal 11βHSD2 activity and the absence of MR expression in the neonatal kidney leave glucocorticoids free to access and activate GR, which might have an important role in kidney development and maturation. It has been suggested that glucocorticoids are implicated in developmental programming [10], [11]. This hypothesis could be sustained by the fact that, at variance with the kidney, 11βHSD2 activity is detected in other organs in the newborn. It is particularly interesting to note that while it is barely expressed in the adult brain, a high 11βHSD2 activity is detected in the developping central nervous system in rats, mice and humans until the end of gestation [24], [44]. It has been postulated that 11βHSD2 during fetal and neonatal life is essential to protect the developing nervous system from deleterious consequences of glucocorticoid exposure [45]. Therefore 11βHSD2 could have an organ specific pattern of expression in the neonatal period, protecting against or facilitating glucocorticoid actions. This developmental process could contribute to the increased short-term adverse outcome rate observed in extremely low birth weight infants with high cortisol concentrations [46]. Moreover, this specific temporal window where the kidney appears to be particularly sensitive to glucocorticoid action could explain the deleterious effects of prenatal glucocorticoid overexposure on renal development [47], [48] or epigenetic modifications leading to the predisposition for adult hypertension [11], [49], [50].

Finally, our study demonstrates the existence of a physiological, temporal 11βHSD2 expression window specific to the kidney, which appears to be necessary for optimal fetal and neonatal development, but as a result could also represent a breach in the protective mechanisms against excess glucocorticoid exposure responsible for adverse short-term and long-term effects through fetal programming, with a higher predisposition to specific diseases in later life.
